# Mucins produced by type II pneumocyte: culprits in SARS-CoV-2 pathogenesis

**DOI:** 10.1038/s41423-021-00714-8

**Published:** 2021-06-09

**Authors:** Bo Huang

**Affiliations:** 1 Department of Immunology & National Key Laboratory of Medical Molecular Biology, Institute of Basic Medical Sciences, Chinese Academy of Medical Sciences (CAMS) & Peking Union Medical College, Beijing, China; 2 Department of Biochemistry & Molecular Biology, Tongji Medical College, Huazhong University of Science & Technology, Wuhan, China

**Keywords:** Viral infection, Alveolar macrophages

The pandemic of severe acute respiratory syndrome coronavirus 2 (SARS-CoV-2) is sweeping across the world and has caused the loss of more than 3.3 million lives. Before clearance by virus-specific T and B cell-mediated adaptive immunity, excessive inflammation by innate immune cells might cause severe lung and even multiorganic pathologies, thus interfering with antiviral immunity. To curb infection and subsequent organic damage, a deep understanding of the pathogenetic process is highly desirable. Recently, we found that IFN-driven mucin expression in type II alveolar epithelial cells is crucial in initiating hypoxia and early lung pathology^1^, and SARS-CoV-2 is disposed of by M1 and M2 alveolar macrophages (AMs) in distinct manners^2^. In this correspondence, we propose that (1) following the invasion of the alveoli and uptake by local alveolar macrophages, SARS-CoV-2 may stimulate macrophages to produce proinflammatory cytokines, including type I interferon; (2) type I interferon acts on neighboring alveolar type II pneumocytes and activates the cytoplasmic transcription factor aryl hydrocarbon receptor (AhR); (3) subsequently, AhR is translocated to the nucleus, where it promotes the expression of mucin genes, leading to mucus production; and (4) mucus begins to accumulate in the alveoli and gradually impairs the exchange of O_2_ and CO_2_, initially causing hypoxia and then dampening CO_2_ exhalation, leading to a critical illness. Here, we dissect these early pathogenic events, which might provide clues to interfering with SARS-CoV-2 infection at an early stage.

## Alveoli are the battlefield

The primary function of the lungs is to inhale oxygen and exhale carbon dioxide. Anatomically, the respiratory trachea branches into two bronchi. The latter further branches into bronchioles and respiratory bronchioles, which end in alveoli. Histologically, bronchial and bronchiolar ciliated cells and brush cells have cilia that discharge particle-trapping mucus that is mainly produced by goblet cells. In contrast, alveoli neither have cilia nor produce mucus under normal conditions. Based on the clinical symptoms of dry cough, it is likely that this virus mainly invades alveoli. Otherwise, cough with sputum should be generated. Consistent with this conclusion, type II pneumocytes highly express ACE2 and can be easily infected by SARS-CoV-2^3^. Thus, the question is what happens to type II pneumocytes following viral invasion.

### Silent hypoxia is explained by alveolar mucus

Hypoxia is a typical symptom of COVID-19. Excessive inflammation caused by viral infection may elevate capillary permeability and even damage the endothelium, leading to the accumulation of alveolar fluid and subsequent hypoxia. In support of this hypothesis, clinical imaging shows ground-glass opacity in the lungs. However, many COVID-19 patients initially have oxygen deprivation without breathing problems, and this enigmatic phenomenon is called silent hypoxia by physicians^4^. This silent hypoxia suggests that early factors trigger hypoxia before excessive acute inflammation. Autopsy revealed copious amounts of gray-white viscous fluid in the lungs of COVID-19 patients, and single-cell RNA-seq analysis showed mucin expression in the lung epithelial cells of these patients^5^. Mucus adhesion and accumulation in the alveoli may increase the thickness of the blood-gas barrier, thus causing hypoxia.

### CO_2_ retention is the key event in switching hypoxia to rapid illness

In the lungs, O_2_ is inhaled from the air, and CO_2_ is generated from tissue cells in which CO_2_ is produced through the tricarboxylic acid (TCA) cycle. As a waste product, CO_2_ is released from cells into interstitial fluid and further diffuses into capillary vessels. Subsequently, CO_2_ is brought to the lungs and expelled by crossing the blood-gas barrier during exhalation. Normally, O_2_ and CO_2_ exchange between alveoli and pulmonary capillary blood is achieved through a passive diffusion process, which can be influenced by the thickness of the blood-gas barrier and the solubility of the gas. Mucus in alveoli adheres to and increases the barrier thickness, thus hindering gas diffusion. Although O_2_ and CO_2_ face the same barrier, CO_2_ has a 20-fold higher solubility than O_2_^6^. Thus, at an early stage, a certain degree of increased mucus might only affect O_2_ but not CO_2_ diffusion. As long as CO_2_ can be expelled, blood pH homeostasis can be maintained, and the mitochondrial TCA cycle can occur normally. Thus, although silent hypoxia occurs at the early stage, COVID-19 patients can be normal with no symptoms. However, once the disease enters a certain stage (such as increased mucus in alveoli, which can impede CO_2_ diffusion), patients illness rapidly progresses.

### IFNs trigger mucus production by type II pneumocytes

Next, the question is how alveolar mucus is generated. Given the infection of type II pneumocytes by the virus, a simple idea is that SARS-CoV-2 infection directly causes alveolar mucus production. However, our study showed that direct infection of alveolar epithelial cells with SARS-CoV-2 did not induce mucin expression by these cells^1^. When SARS-CoV-2 invades the alveoli, AMs wait there and can recognize and respond to viral PAMPs, leading to the generation of interferons, which exert crucial antiviral immunity. Unexpectedly, we found that both IFN-β and IFN-γ effectively induce mucin expression in type II pneumocytes through the IDO-kynurenine-AhR pathway^1^. This mucin production induced by IFNs might be an evolutionary response to protect alveolar epithelial cells that, unfortunately, might cause damage.

### AMs are potential sources of early IFN production

Thus, the cellular source of IFNs remains unclear. IFN-γ is mainly produced by T cells. However, IFN-β can be produced by almost all cell types, especially by plasmacytoid DCs, upon viral infection. In the case of SARS-CoV-2 infection, AMs might be the key producers of IFNs during early infection. After entering the alveoli, SARS-CoV-2 can be quickly taken up by AMs. Thus, the activated PAMP-PRR system results in the production of IFN, which then acts on local type II pneumocytes, leading to mucus production. Indeed, we found that large amounts of IFNs are produced by AMs following SARS-CoV-2 infection^2^. Intriguingly, the virus can be amplified in AMs depending on their phenotype^2^.

**Fig. 1:**
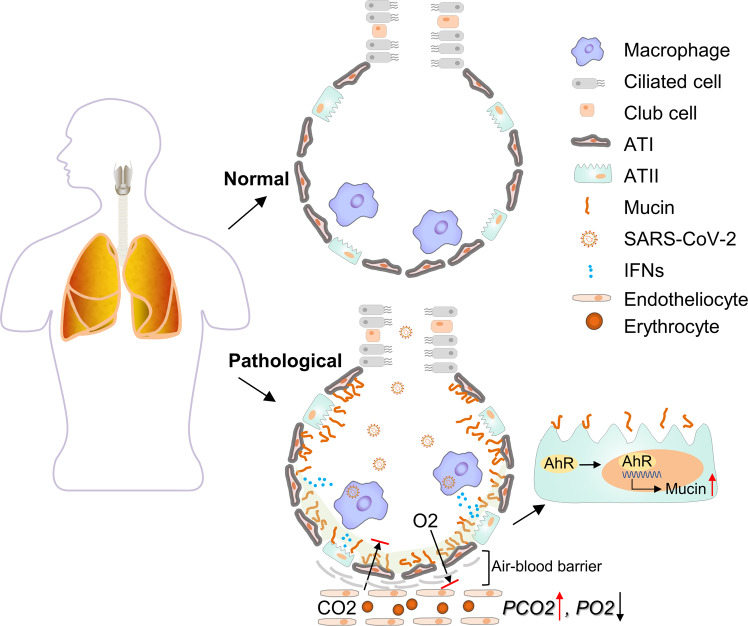
Schematic showing IFN-induced upregulation of mucins in hypoxia in COVID-19 patients. (1) Air inhalation brings SARS-CoV-2 into the alveoli; (2) alveolar macrophages recognize the invading virus and respond by releasing IFN-β; (3) other innate immune cells, such as plasmacytoid DCs and γδ T cells, may also respond to the virus and produce IFNs, including IFN-β and IFN-γ; (4) type II pneumocytes are mainly stimulated by locally released IFN-β, thus activating the IDO1-Kyn-AhR signaling pathway; (5) activated AhR transcriptionally promotes the expression of mucin genes; (6) the generated mucus adheres to the surface of alveoli and impairs oxygen entrance into the blood but not CO_2_ entrance into alveoli, leading to silent hypoxia; (7) mucus further accumulates in the alveoli, and proinflammatory factors in the alveoli stimulate capillary vessels to allow the leakage of blood, thus leading to the hindrance of CO_2_ exchange; and (8) mucus further accumulates in the alveoli, while proinflammatory factors become critical.

Based on these analyses, we outlined the early events following SARS-CoV-2 infection in Fig. 1. We suggest that the production of mucins by type II pneumocytes is a trigger for silent hypoxia and that impaired CO_2_ exhalation is the turning point at which patients shift from silent hypoxia to critical illness.

## Supplementary information

merged manuscript
