# Supplementary material for: Mucins produced by type II pneumocyte: culprits in SARS-CoV-2 pathogenesis
Source: Cell Mol Immunol. 2021 Jun 9;18(7):1823–5. doi: 10.1038/s41423-021-00714-8 (PMC8188152; doi:10.1038/s41423-021-00714-8)
Supplement: Supplementary file 1 — merged manuscript [file 41423_2021_714_MOESM1_ESM.pdf]

Mucins by type II pneumocytes: culprit for SARS-CoV-2 pathogenesis

Bo Huang[1,2]

[1]Department of Immunology & National Key Laboratory of Medical Molecular Biology, Institute of Basic Medical Sciences, Chinese Academy of Medical Sciences (CAMS) & Peking Union Medical College, Beijing 100005, China

[2]Department of Biochemistry & Molecular Biology, Tongji Medical College, Huazhong University of Science & Technology, Wuhan 430030, China.

Correspondence: tjhuangbo@hotmail.com

The pandemic of severe acute respiratory syndrome coronavirus 2 (SARS-CoV-2) is sweeping across the world and has caused the loss of more than 3.3 million lives. Before the clearance by virus-specific T and B cells-mediated adaptive immunity, excessive inflammation by innate immune cells might cause severe lung and even multi-organic pathologies, thus interfering with the antiviral immunity. To curb the infection and the subsequent organic damage, a deep understanding of the pathogenetic process is highly desirable. Recently, we found that IFNs-driven mucin expression in type II alveolar epithelial cells is crucial to initiate hypoxia and early lung pathology,[1] and SARS-CoV-2 is disposed of by M1 and M2 alveolar macrophages (AMs) in distinct manners.[2] In this correspondence, we propose that (1) following the invasion of the alveoli and the uptake by local alveolar macrophages, SARS-CoV-2 may stimulate the macrophages to produce proinflammatory cytokines, including type I interferon; (2) type I interferon acts on neighbor alveolar type II pneumocytes and activates the cytoplasmic transcription factor AhR; (3) subsequently, AhR is translocated to the nucleus where it promotes the expression of mucin genes, leading to mucus production; (4) mucus is beginning to accumulate in the alveoli and gradually impairs the exchange of O_2 and CO_2, initially causing hypoxia and then dampening CO_2 exhalation, leading to a critical illness. Here, we dissect these early pathogenic events which might provide clues to interfere with SARS-CoV-2 infection at an early stage.

1. Alveoli are the battlefield

The primary function of the lungs is to inhale oxygen and exhale carbon dioxide. Anatomically, the respiratory trachea branches off into two bronchi. The latter further branch off into bronchioles and respiratory bronchioles, which end in alveoli. Histologically, bronchial and bronchiolar ciliated cells and brush cells have the cilia that discharge particle-trapping mucus mainly produced by goblet cells. In contrast,

the alveoli neither have cilia nor produce mucus under a normal condition. Based on the clinical symptom of dry cough, it is likely that this virus mainly invades alveoli. Otherwise, cough with sputum should be generated. In line with this, type II pneumocytes highly express ACE2 and can be easily infected by SARS-CoV-2.[3] Thus, the question is what happens to type II pneumocytes following the viral invasion?

2. Silent hypoxia is explained by alveolar mucus

Hypoxia is a typical symptom of COVID-19. Excessive inflammation caused by viral infection may elevate capillary permeability and even damage endothelium, leading to the formation of alveolar fluids and subsequent hypoxia. In support, clinical imaging displays the ground-glass opacity of the lungs. However, many COVID-19 patients initially have oxygen deprivation without breathing problems, and this enigmatic phenomenon is called silent hypoxia by physicians.[4] This silent hypoxia implies that earlier factor(s) triggers hypoxia before excessive acute inflammation. The autopsy revealed copious amounts of a gray-white viscous fluid in the lungs of COVID-19 patients and single-cell RNA-seq analysis showed mucin expression in lung epithelial cells of the patients.[5] The mucus adherence and accumulation in the alveoli may increase the thickness of the blood-gas barrier, thus causing hypoxia.

3. CO_2 retention is the key event for switching hypoxia to rapid illness

In the lungs, O_2 is inhaled from air but CO_2 is generated from tissue cells where CO_2 is produced through the tricarboxylic acid (TCA) cycle. As a waste product, CO_2 is released from cells to the interstitial fluid and further diffuses into capillary vessels. Subsequently, the CO_2 is brought to the lungs and expelled out by crossing the blood-gas barrier during exhalation. Normally, O_2 and CO_2 exchange between

alveoli and pulmonary capillary blood is achieved through a passive diffusion process, which can be influenced by the thickness of the blood-gas barrier and the solubility of gas. Mucus in the alveoli adheres to and increases the barrier thickness, thus hindering gas diffusion. Although O_2 and CO_2 face the same barrier, the fact is that CO_2 has 20-fold higher solubility than O_2.[6] Thus, at an early stage, a certain degree of increased mucus probably might only influence O_2 but not CO_2 diffusion. As long as CO_2 can be expelled, the blood pH homeostasis can be maintained and the mitochondrial TCA cycle can be normally processed. Thus, although the silent hypoxia at the early stage occurs, COVID-19 patients can be normal with no symptom. However, once the disease enters a certain degree (such as more mucus in alveoli which can impede CO_2 diffusion), the illness of the patients becomes serious rapidly.

4. IFNs are the culprit to trigger mucus production by type II pneumocytes

Next, the question is how alveolar mucus is generated. Given the infection of type II pneumocytes by the virus, a simple idea is that SARS-CoV-2 infection directly causes alveolar mucus production. However, our study found that the use of SARS-CoV-2 to directly infect alveolar epithelial cells did not induce mucin expression by the cells.[1] When SARS-CoV-2 invades the alveoli, AMs are waiting there and can recognize and respond to viral PAMPs, leading to the generation of interferons to exert crucial antiviral immunity. Unexpectedly, we found that both IFN-β and IFN-γ effectively induce mucin expression in type II pneumocytes through an IDO-kynurenine-AhR pathway.[1] This mucin production by IFNs might be an evolutionary result in order to protect alveolar epithelial cells, which, however, might be kind enough to do bad things.

5. AMs are the potential source for early IFN production

Then, the question is about the cellular source of IFNs. It is well known that IFN-γ is mainly produced by T cells. However, IFN-β can be produced by almost all cell types, especially by plasmacytoid DCs upon viral infection. In the case of SARS-CoV-2 infection, AMs might be the key producer of IFNs during the early infection. Once entering the alveoli, SARS-CoV-2 can be quickly taken up by AMs. Thus, the activated PAMP-PRR recognition system results in IFN production, which then acts on local type II pneumocytes, leading to mucus production. Indeed, we found that a large amount of IFNs is produced by AMs following SARS-CoV-2 infection[2]. Intriguingly, the virus can be amplified in AMs dependent on their phenotype[2].

Based on the above analyses, we draw an outline of the early events following SARS-CoV-2 infection in Figure 1. We suggest that the production of mucins by type II pneumocytes is a triggering point for silent hypoxia and CO_2 expel impairment is the turning point for the patients from silent hypoxia to critical illness.

ACKNOWLEDGMENTS

This work was supported by the National Natural Science Foundation of China (81788101) and Chinese Academy of Medical Sciences (CAMS) Initiative for Innovative Medicine (2020-I2M-CoV19-007).

DECLARATION OF INTEREST

The author declares no competing financial interests.

REFERENCES

1 Liu, Y. et al. Mucus production stimulated by IFN-AhR signaling triggers hypoxia of COVID-19. *Cell Res*, 1-10 (2020).

2 Lv, J. et al. Distinct uptake, amplification, and release of SARS-CoV-2 by M1 and M2 alveolar macrophages. *Cell Discov* **7**, 1-24 (2021).

3 Lv, J. et al. ACE2 expression is regulated by AhR in SARS-CoV-2-infected macaques. *Cell Mol. Immunol* **18**, 1308-1310 (2021).

4 Tobin, M. J., Laghi, F. & Jubran, A. Why COVID-19 Silent Hypoxemia Is Baffling to Physicians. *Am. J. Respir. Crit. Care. Med* **202**, 356-360 (2020).

5 He, J. *et al.* Single-cell analysis reveals bronchoalveolar epithelial dysfunction in COVID-19 patients. *Protein & cell* **11**, 680-687 (2020).

6 Wagner, P. D. The physiological basis of pulmonary gas exchange: implications for clinical interpretation of arterial blood gases. *Eur. Respir. J* **45**, 227-243 (2015).

FIGURE LEGEND

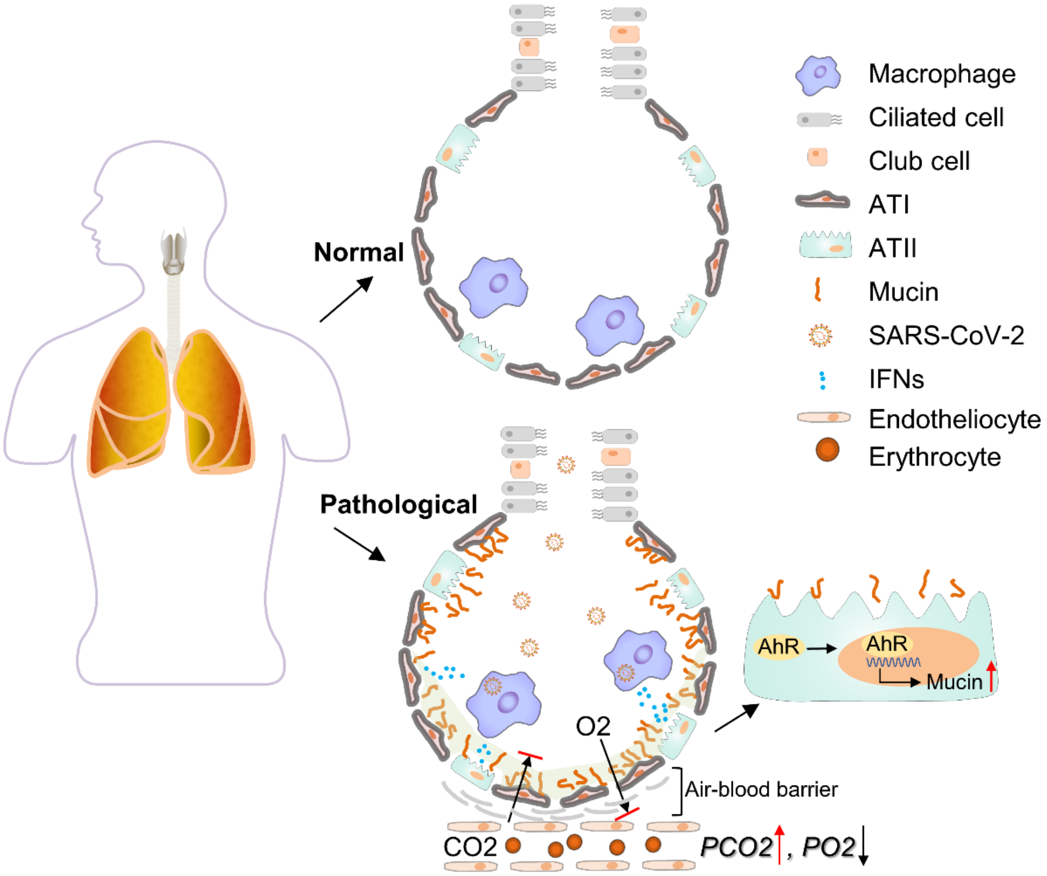

Fig. 1 A schematic for IFNs-upregulated mucins in the hypoxia of COVID-19 patients. (1) air inhalation brings SARS-CoV-2 into the alveoli; (2) alveolar macrophages recognize the invaded viruses and make a response by releasing IFN-β; (3) other innate immune cells such as plasmacytoid DCs and γδ T cells may also respond to the viruses and produce IFNs, including IFN-β and IFN-γ; (4) type II pneumocytes are mainly stimulated by locally released IFN-β, thus activating the IDO1-Kyn-AhR signaling pathway; (5) activated AhR transcriptionally promotes the expression of mucin genes; (6) the generated mucus adheres to the surface of alveoli and impairs oxygen entering the blood but not CO_2 entering the alveoli, leading to silent hypoxia; (7) mucus is further accumulated in the alveoli and proinflammatory factors

at the alveolar site stimulate capillary vessels to allow the leakage of blood, thus together leading to the hindrance of CO_2 exchange; (8) once CO_2 exchange is impeded, the illness rapidly switches to a critical state. Here,
